# Plant density and life history traits of *Aconitum spicatum* in North-central Nepal: effects of elevation and anthropogenic disturbances

**DOI:** 10.7717/peerj.7574

**Published:** 2019-09-10

**Authors:** Deep J. Chapagain, Henrik Meilby, Suresh K. Ghimire

**Affiliations:** 1 Central Department of Botany, Tribhuvan University, Kathmandu, Nepal; 2 Department of Food and Resource Economics, University of Copenhagen, Copenhagen, Denmark

**Keywords:** Population density, Tuber biomass, Sensitivity, Mixed Zero Inflated Poisson

## Abstract

Increasing cross-border trade of medicinal and aromatic plants (MAPs) has put heavy pressure on a considerable number of species in the Himalayas. One of the threatened species in Nepal is *Aconitum spicatum*. Unfortunately for this species and for many others, our knowledge on population ecology and performance across the distribution range is insufficient, hindering the formulation of species-specific management plans. We therefore studied density and population structure of *A. spicatum* and assessed variation in its life history traits among three populations (subalpine, lower alpine and alpine) along an elevation gradient (3,000–4,200 m a.s.l.) in Annapurna Conservation Area, north-central Nepal. The results show that human disturbances and topographic factors contributed to the variation in density and life history traits. The overall density ranged between 0.56 ± 0.09 (Mean ± SE) and 2.48 ±  0.24 plants/m^2^ with highest mean density in the lower alpine and lowest in the subalpine population. The subalpine population was also characterized by lower investment in reproductive structures with lowest seed mass and low seed viability and fecundity. Among the environmental variables tested, harvesting, animal droppings and fire appeared to be the most important factors affecting density of different life stages of *A. spicatum*. The prevailing harvesting pattern is destructive as it involves uprooting of the whole plant and this appears to be a main reason for low recruitment and reduced density of the subalpine population. The level of disturbance decreased with increasing elevation. In terms of reproductive effort, the alpine population performed best. Our results indicate that the viability of *A. spicatum* populations depends on controlling over-harvesting and pre-mature harvesting of tubers and protecting younger life stages from grazing, trampling and fire. We therefore recommend that when formulating management guidelines, measures aiming to mitigate such anthropogenic disturbances should be considered.

## Introduction

The study of variation in life history traits of a species is crucial to understanding the role of particular types of ecological pressure in shaping population demographics (Roff, 1992; Réale et al., 2010; Tonnabel et al., 2017). Variation in environmental conditions can affect recruitment dynamics (Franco & Silvertown, 1996) and population growth rates (Stearns & Hoekstra, 2000; Caswell, 2001). Himalayan alpine plants respond to environmental and climate change variables including biogeography, elevation and precipitation (Salick et al., 2014). Increasing anthropogenic activities have also created and continue to create a range of novel environments in mountain ecosystems (Zhang et al., 2019). Along the elevation gradient in mountain ecosystems, large changes in environmental factors occur over short distances, leading to huge variation in the selection pressure imposed on plant life-history strategies and traits (Bresson et al., 2011). Particularly, topographic variation affects microclimatic conditions, which may contribute to variation in life history traits. These variations are coupled with disturbance regimes, like harvesting, fire and grazing which themselves are structured along elevation gradients. The concentration of these anthropogenic disturbances, especially in alpine and subalpine ecosystems may put high-altitude plants under stress. To cope with such stressful conditions, high-altitude plants develop self-sustaining adaptation strategies (Körner, 2003), including a long floral axis, more globular flowers (Molau, 1993), persistent sepals, larger root systems (Körner, 1999), vegetative apices or perennating buds at safe depth below ground (Körner, 2003), higher proportion of belowground biomass (Webber & May, 1977) and increased number and size of seeds (Körner, 1999), which contribute to enhance the persistence of their populations.

Population density and individual-level traits, like plant size and reproductive performance (Totland & Nyléhn, 1998; Nielsen, Totland & Ohlson, 2007; Fan & Yang, 2009) are considered the most important basic parameters needed to assess the capacity of a plant species to maintain its population. Reproductive traits are especially important for a species to establish and to persist following disturbance. Rates of recruitment vary and depend on, e.g., seed number, seed mass, seed viability, growing season, and disturbance characteristics (Chambers, MacMahon & Brown, 1990). Understanding the variation in such traits is important for the assessment of the long-term persistence of populations growing in habitats disturbed by human activities. Habitat disturbance often has negative effects on plant reproductive success (Chambers, 1995). Cattle grazing, trampling and fire, for example, may lead to lower fruit set and subsequent recruitment (McKechnie & Sargent, 2013; Renison et al., 2015). However, fire is deemed to be a prerequisite for recruitment, growth and development of plant species in some ecosystems (Bucini & Lambin, 2002) and has in some cases been shown to promote higher species diversity (Hobbs, Mallik & Gimingham, 1984).

Harvesting of medicinal and aromatic plants (MAPs) is a customary subsistence practice in the Himalayan region. Over 300 taxa of MAPs are traded from Nepal with a total annual amount of 10,770 tons and a value of USD 60.09 million (Ghimire et al., 2016; Government of Nepal, 2015; Government of Nepal, 2018). Trans-boundary trade of Himalayan MAPs has increased to meet the growing demand from international pharmaceutical companies and has led to heavy and indiscriminate harvesting of some species. *Aconitum spicatum* (family Ranunculaceae) is among the ten most traded medicinal plants from the Himalayan region of Nepal (Olsen & Larsen, 2003). It is vulnerable in Nepal, mainly due to over-harvesting (Bhattarai, Tandon & Ved, 2002). Considering the high trade value of *A. spicatum*, the government of Nepal has nominated it as one of thirty herbs of national priority for development, research and cultivation (Department of Plant Resources, 2012). Despite the commercial interest and high conservation value of *A. spicatum*, detailed studies describing its ecological, morphological and reproductive characteristics are largely lacking within its entire range. Scientific information on how the different disturbance regimes (including harvesting) shape the life history parameters (reproduction and growth) and determine population density and structure of important Himalayan MAPs like *A. spicatum* across ecological gradients is important for the development of management strategies to maintain viable populations (Ticktin, 2004; Ghimire, McKey & Aumeeruddy-Thomas, 2005). In this paper, we aim to analyze whether impacts of anthropogenic disturbance on population performance of *A. spicatum* depend on ecological factors associated with elevation. Thus, examining populations located within three consecutive elevation ranges, the goal of this study was to answer the following questions: (i) do the impacts of anthropogenic disturbance on population structure and density of *A. spicatum* vary along an elevation gradient? (ii) How do plant size and reproductive performance of *A. spicatum* vary along an elevation gradient?

## Materials and Methods

### Study area

**Figure 1:**
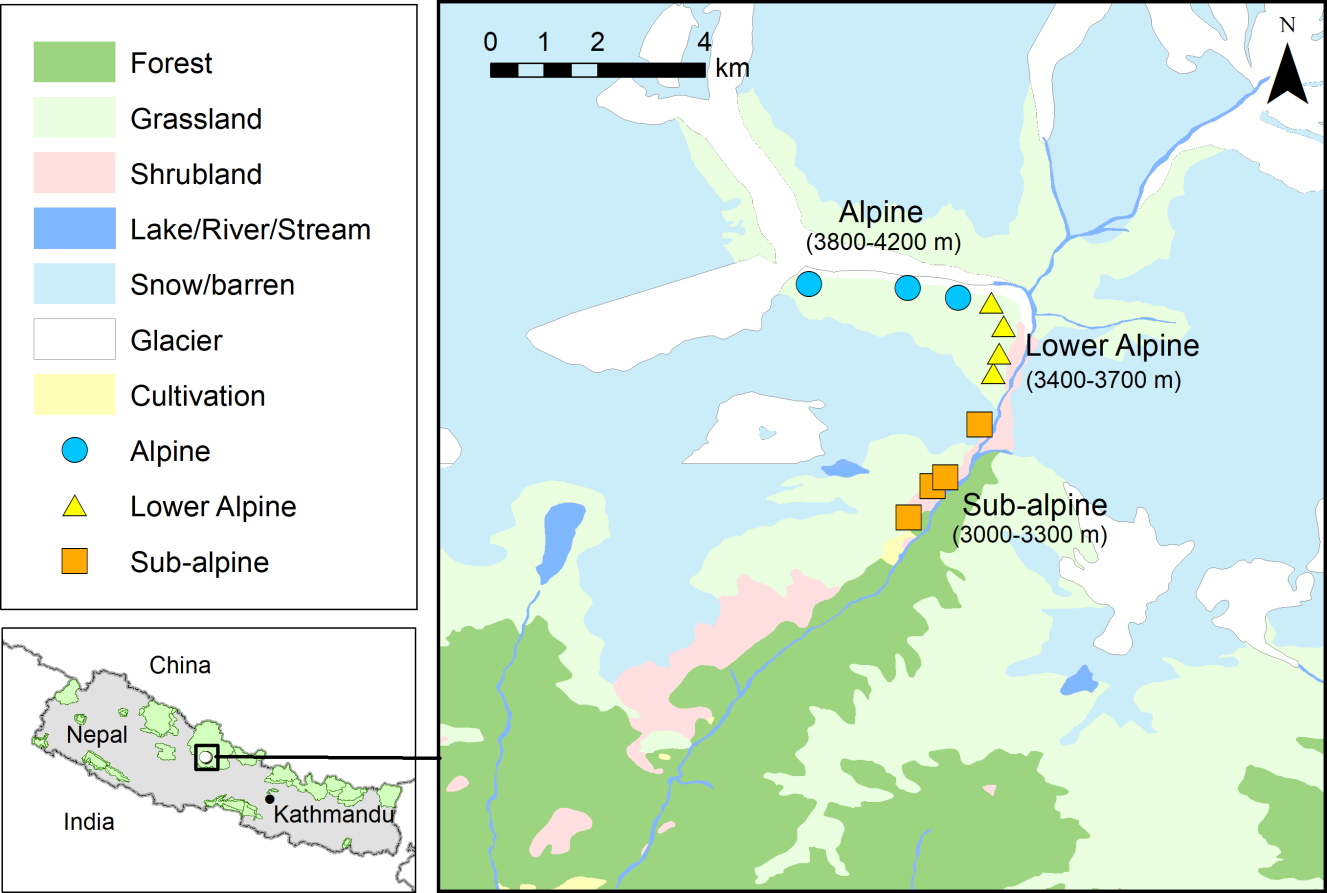
Map showing study area (N28°29.455′ and E083°53.546′ to N28°31.806′ and E083°52.537′, Annapurna Conservation Area) and the locations of the *Aconitum spicatum* populations sampled. Source of land cover map: Department of Survey, Government of Nepal.

The study was carried out in upper Modi River Valley (N28°29.455′and E083°53.546′to N28°31.806′and E083°52.537′) within Annapurna Conservation Area (ACA) in north-central Nepal (Fig. 1). The study area is characterized by temperate and alpine climate. The vegetation consists of upper temperate coniferous and mixed broad-leaved forests at lower elevations (>3,000–3,500 m), sub-alpine mixed forests dominated by *Abies spectabilis* and *Betula utilis* at and below the tree line (>3,500–4,000 m), lower alpine thickets mainly of dwarf bushes of *Rhododendron* spp. at and above the tree line (*ca*. 4,000 m), and alpine meadows and grasslands above 4,000 m. Snowfall starts in October and the area remains snow-covered for up to six months, until March. High seasonal differences in temperature and rainfall, short growing season and heavy snowfall are the main constraints to plant growth at higher elevations. The study area comprises the most popular trekking routes of Nepal, and each year approximately 30,000 tourists visit the area. Trekking tourism can be considered one of the most important factors affecting the structure of forest ecosystems at high elevation in the Himalayan region (Biønness, 1980; Stevens, 2003; Garbarino et al., 2014). There are 14 hotels along the trekking route passing through the study area, and the nearest villages (Chhomrong and Sinwa) are inhabited by 62 households. Along the upper Modi Valley there are two cattle sheds (’goths’ in Nepali), each with about 500 cattle. The livelihood of local people is based on subsistence agriculture, pastoralism, seasonal trade, and more importantly, tourism. Interviews with local residents indicated that people from the neighboring Gorkha and Dhading districts of Nepal, recently started harvesting highly valued MAPs, including *A. spicatum* from the ACA region (D Chapagain, pers. obs., 2018).

### Study species

*A. spicatum* is a vulnerable (Bhattarai, Tandon & Ved, 2002) medicinal plant species with a distribution restricted to the Himalayas of Nepal, India, Bhutan and China, at elevations between 2,900 and 4,200 m above sea level (asl) (Ghimire et al., 1999). In the study area, fragmented populations of *A. spicatum* occur between 3,000–4,200 m asl. It is a tall perennial herb with an erect stem reaching up to two m in height and bearing a crown of blue zygomorphic flowers with numerous stamens. It bears 1–70 flowers (mean ± SE 10.64 ± 1.29) and 1–110 fruits (mean ± SE 14.03 ± 1.53) per plant. Each fruit is an aggregate of five to six follicles. Each fruit produces an average of 41 seeds; thus an individual plant bears about 574 seeds on average. The plant produces paired dark-brown tubers, which are best harvested as soon as the above-ground parts die in autumn (Ghimire et al., 1999). The aerial part of the plant is annual but the tubers are perennial. The mother tuber dies every year, but before this happens it produces a number of sprouting buds, which grow into one to five daughter tubers (direct field observation). The plant shows low seed germination potential and the seeds can stay dormant for a long time (Srivastava et al., 2010), and even tubers show frequent dormancy (D Chapagain, pers. obs., 2018). The plant is highly poisonous, and the tubers contain alkaloids, mainly aconitine, hypaconitine and mesaconitine, which are reported to have antibacterial, antipyretic, analgesic and enzyme inhibition properties (Srivastava et al., 2010). Tubers of *A. spicatum* are used in traditional medicines to cure fever, headache and infections in lungs and intestines and to heal cuts and wounds (Tsarong, 1994; Lama, Ghimire & Aumeeruddy-Thomas, 2001).

### Sampling

This research is part of a long-term study carried out between 2015 and 2018 to understand the population dynamics of *A. spicatum* in ACA (research permit issued by Department of National Park and Wildlife Conservation (DNPWC), ref. No. 93/070/071). During the summer of 2015, sampling was carried out in three sites at elevations ranging from 3,000 m to 4,200 m. Sampling covered the whole elevation range of *A. spicatum* in the study area as the transects were established from the lowest to the highest point of its distribution. In each 100 m elevation band, a transect with six plots (three m × three m) was established with plots located at a minimum plot-to-plot distance of 10 m. Each plot was divided into nine subplots of one m × one m, five of which (center and four corner subplots) were systematically selected for detailed biological measurements. In total, we laid out 66 (three m × three m) plots, with 330 (one m × one m) sub-plots. In each plot, we recorded latitude and longitude using an eTrex Garmin GPS device and cross-checked the elevation using a calibrated altimeter. Slope and aspect were measured using clinometer and compass, respectively. In each subplot, the cover (%) of shrubs, herbs, grasses, lichens, bryophytes, litter, bare ground, solid rock and scree were estimated using standard methods (Pauli et al., 2015).

Individuals of *A. spicatum* were classified into four stage classes on the basis of their size and reproductive status and were counted. The stage classes were: seedlings (Sd; stem girth ≤ one mm measured at five cm from soil surface, leaf number = 1–2), juveniles (Jv; stem girth = 1–<three mm, leaf number ≥ 2–<5), vegetative adults (Adv; stem girth ≥ three mm, leaf number ≥ 2) and reproductive adults (Adr; flowering or fruiting individuals). In each subplot, the count of plants at different stages was used to calculate population structure and density. Animal droppings, trampling, harvesting and fire were considered as anthropogenic disturbances. A five-point ordinal scale (0–4) was applied to describe the level of disturbance. A score of ‘0’ corresponded to no disturbance, while ‘4’ corresponded to heavy disturbance.

We grouped four elevation bands together and defined three wider ecotone populations, namely subalpine (3,000–3,300 m), lower alpine (3,400–3,700 m) and alpine (3,800–4,200 m). We uprooted twenty of the most vigorous plants from each of these populations to record plant biomass and to measure vegetative traits (plant height, stem girth, number of leaves, leaf size and length of floral axis) and reproductive traits (number of buds, flowers and fruits). The fresh weight of aboveground and belowground plant parts was measured in the field. Afterwards, the plants were air dried, packed in paper bags and brought to the laboratory for dry biomass estimation. Seed number and seed mass estimations were based on random selection of 15 mature reproductive individuals from each population during the seed maturation period in October. From each such individual, we collected five fruits, recorded their diameter, length and fresh weight in the field, and then packed the fruits separately in paper bags for subsequent dry biomass estimation.

We carried out seed sowing experiments in October 2015 and 2016. In each year, we introduced mature seeds (*n* = 100) into each of five randomly selected one m × one m subplots, located in a similar habitat adjacent to the study plots. Dark brown seeds which sank in water were considered mature and were selected for the sowing experiment. The seeds sown during 2015 failed to germinate. However, the seeds sown in 2016 germinated and established in 2017. Seedlings that recruited and survived were recorded in 2017. The data resulting from these experiments along with the information on seed production were used to estimate fecundity. Fecundity was calculated as the percentage of established seedlings from 100 seeds multiplied by the average number of viable seeds per individual in each population.

### Laboratory study

We oven dried (at 60 °C for 72 h) all the air-dried plant materials and measured their biomass to an accuracy of 0.001 g. Similarly, we counted the number of seeds per fruit and per individual plant, and measured the seed mass (expressed as dry mass of 50 seeds) per individual. We tested seed viability by soaking the seeds in water, cutting them into halves and subsequently dipping them in a 10 percent solution of 2, 3, 5 Triphenyl Tetrazolium Chloride (TTC) for a minimum of 12 h in total darkness. We examined pink coloration of the embryo under a microscope, indicating viability of the seed (Beigh, Nawchoo & Iqbal, 2006).

## Statistical analysis

### Population structure and density

Given the clustered sampling design with five sub-plots per plot, we analyzed the variation of *A. spicatum* density using a mixed-modeling approach including a random effect of plot. Over-dispersion of the count data was confirmed by using the qcc package (Scrucca, 2004), and the analysis of the counts for different stages was made using the glmmTMB package (Mollie et al., 2017) in R, version 3.5.3 (R Development Core Team, 2017).

Direct field observations confirmed the plant as rare and sparsely distributed at the study site. Hence, methods especially designed to cater for count data with excess zeros were considered from the project’s early stages. Simple transformations, such as square root and log, were not capable of making our data appropriate for modeling using standard statistical assumptions of normality. The abundance of zero counts prevented these transformations from having the desired effect of symmetrizing the data and eliminating dependence between the mean and variance. Based on the Akaike Information Criterion (AIC), the best fit among several model alternatives was obtained using Mixed Zero-Inflated Poisson (mixed ZIP) models (Hall, 2000). Hence, we used mixed ZIP models to quantify the relationship between environmental factors and the abundance of plants at different stages of development using explanatory variables such as population (a given elevation range), relative radiation index (RRI), cover of shrubs, cover of herbs, harvesting, trampling, animal dropping and fire. All models included a random effect of the plot. We prepared twenty-six sets of candidate models using the glmmTMB package (see Appendix S1) and finally prepared an average model based on a set of 16 best candidate models (selected on the basis of delta AIC) using the MuMIn package (Barton, 2018) in R. The 16 models with AIC values less than 258.4 for seedling stage, 330.9 for juvenile stage, 583.3 for adult vegetative stage and 777.3 for adult reproductive stage were used to prepare the average model by MuMIn (see Appendix S2).

The full models were in all cases expressed as:

Density_ij_ (of a particular stage of *A. spicatum*) = a + b (Population) + c_1_ RRI_ij_ + c_2_ Herb cover_ij_  + c_3_ Shrub cover_ij_  + c_4_ Harvesting_ij_  + c_5_ Animal dropping_ij_ + c_6_ Trampling_ij_  + c_7_ Fire_ij_ where, a, b(Population) and c_1_ … c_7_ are fixed model parameters, *i* = 1…66 is the plot (included as a random effect) and j=1…5 is the sub-plot. Population had three categories (subalpine, lower alpine and alpine). Density was expressed as the number of individuals in a given stage category counted within a one m^2^ sub-plot. A relative radiation index (RRI) was used as a proxy of microclimate at the site. RRI is a relative measure of the exposure to solar radiation at noon at a specific location and was calculated for each plot as a function of aspect, latitude, and slope. RRI was calculated as: RRI = cos(180°-Ω) × sin(β) × sin(Φ) + cos(β) × cos(Φ), where Ω = aspect (slope azimuth in degrees), Φ = latitude (degrees) and *β* = slope inclination (degrees) (Oke, 1987; Vetaas, 1992). The preliminary analysis showed that the shrub, litter, bryophyte and lichen cover were closely correlated. Similarly, herb and grass cover, and scree and bare ground cover were closely correlated. It was therefore decided to exclude litter, bryophyte, lichen, grass and scree cover in subsequent analyses.

### Plant size and reproductive performance

Unlike plant density and structure, other life history traits were studied using sample individuals selected at the population level (see above). As the measurements were done on individual plants, we sampled 20 replicates for each of the size-based and reproductive trait variables. Before doing the analysis we examined whether data for each of these variables met the assumptions of parametric tests. Except for plant height and biomass, no other variables met the standard assumptions of normality and homogeneity of variance. Therefore, we applied Kruskal–Wallis tests to examine the variation of these traits among the three populations. In the case of plant height and biomass, logarithmic transformation of the data gave statistical normality, so in these cases we applied one-way ANOVA. A double logarithmic allometric model was prepared to describe the relationship between plant height and the biomass of tubers. We further performed multiple comparison tests after ANOVA and Kruskal Wallis to compare the significant differences among the populations. All tests were conducted using the R version 3.5.3 (R Development Core Team, 2017).

## Results

### Population structure and density

**Figure 2:**
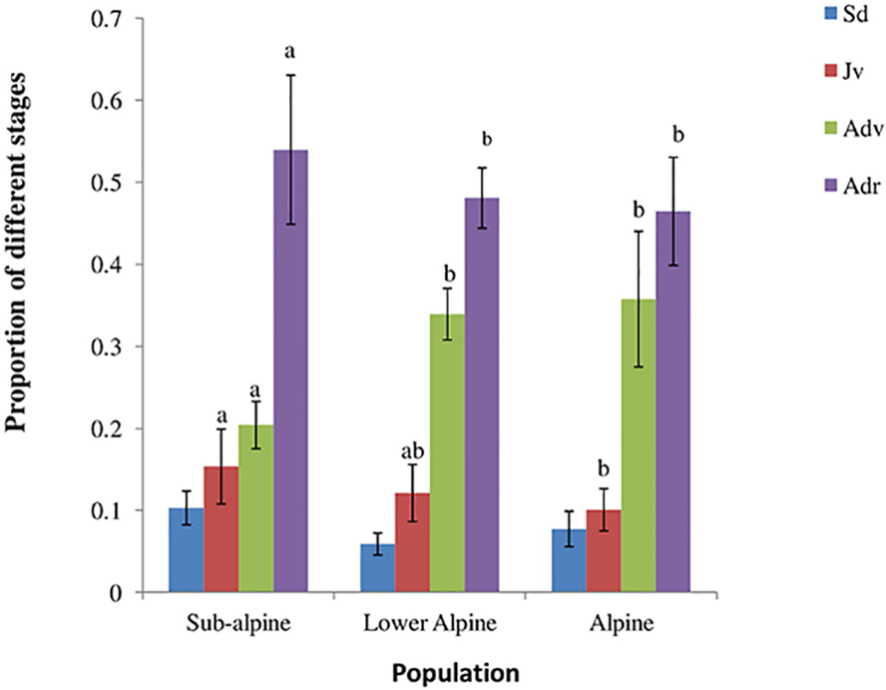
Population structure of *A. spicatum* in Annapurna Conservation Area, Central Nepal (Sd, seedlings; Jv, juveniles; Adv, adult vegetative; Adr, adult reproductive). Bars with same letter for each stage class among populations do not vary significantly at *p* = 0.05. Sd (ns).

We found significant differences in the densities of juvenile, and vegetative and reproductive adults among the three populations (Table 1). Similarly, we found differences in the observed stage distributions among them (Fig. 2). All the populations revealed lower proportions of plants in smaller size classes (seedlings and juveniles) than in larger size classes (vegetative and reproductive adults). The variation in density of *A. spicatum* across elevations was hump-shaped and density reached its maximum in the lower alpine population with a mean density of 2.48 ± 0.24 plants/m^2^. The lowest density (0.56 ± 0.09 plants/m^2^) was found in the subalpine population.

**Table 1:** Population density (m^−2^) for different life stages of *A. spicatum* in three populations in Annapurna Conservation Area, Central Nepal. Densities are stated as mean ± SE. *χ*^2^ and *p*-values were based on Kruskal–Wallis test, *df* = 2, *n* = 66. Values for each stage class among the populations with same superscript letter do not vary significantly at *p* = 0.05 level based on multiple comparison test after Kruskal Wallis.

Population	Seedling	Juvenile	Adult vegetative	Adult reproductive	Total
Subalpine (3,000–3,300 m)	0.05 ± 0.02	0.07 ± 0.03^a^	0.10 ± 0.03^a^	0.34 ± 0.06^a^	0.56 ± 0.09
Lower alpine (3,400–3,700 m)	0.13 ± 0.03	0.30 ± 0.06^b^	0.81 ± 0.12^b^	1.24 ± 0.13^b^	2.48 ± 0.24
Alpine (3,800–4,200 m)	0.2 ± 0.06	0.24 ± 0.13^b^	0.78 ± 0.21^b^	0.93 ± 0.17^b^	2.16 ± 0.48
*χ*^2^ value	5.18	10.33	34.79	34.17	45.23
*p*-value	0.08	<0.01	<0.0001	<0.0001	<0.0001

### Variation in plant size and reproductive performance

The plants showed the greatest vegetative vigor in terms of plant height and stem girth in the subalpine population (Table 2). Similarly, the subalpine population showed the highest total numbers of flowers and fruits per plant (Table 2).

**Table 2:** Variation in life history traits (vegetative traits and reproductive outputs) of *A. spicatum* among three populations in Annapurna Conservation Area, Central Nepal. Values are mean ± SE (*n* = 60). ^∗^Population comparisons were based on either ^1^Kruskal–Wallis tests or ^2^one-way ANOVA. Values for each trait among the populations with same superscript letter do not vary significantly at *p* = 0.05 level based on multiple comparison tests after^1^Kruskal Wallis and ^2^ANOVA.

Traits	Subalpine (3,000–3,300 m)	Lower alpine (3,400–3,700 m)	Alpine (3,800–4,200 m)	*χ*2∕*F* value*	*P*-value
Leaf number^1^	12.05 ± 0.33^a^	10.9 ± 0.46^a^	6.35 ± 0.18^b^	40.22	<0.001
Plant height (cm)^2^	156.43 ± 5.62^a^	151.28 ± 5.29^a^	81.77 ± 4.20^b^	67.47	<0.001
Stem girth (mm)^1^	10.04 ± 0.95^a^	9.60 ± 0.68^a^	6.62 ± 0.25^b^	12.51	0.001
Length of floral axis (cm)^1^	19.67 ± 0.86^a^	19.13 ± 1.42^a^	10.01 ± 0.60^b^	33.18	<0.001
Average leaf area (cm^2^)^1^	175.36 ± 14.21^a^	190.98 ± 10.98^a^	91.57 ± 4.95^b^	31.26	<0.001
No. of buds per plant^1^	29.77 ± 10.03^a^	9.14 ± 2.89^ab^	6.00 ± 1.19^b^	12.48	0.001
No. of flowers per plant^1^	15.85 ± 4.44	10.61 ± 2.03	6.20 ± 0.71	5.29	0.070
No. of fruits per plant^1^	22.15 ± 6.80^a^	17.5 ± 4.56^a^	4.12 ± 0.67^b^	10.72	0.004
Total reproductive parts per plant^1^	39.85 ± 7.31	26.75 ± 5.07	16.45 ± 1.71	4.89	0.086
Number of daughter tubers per plant^1^	1.6 ± 0.19	1.85 ± 0.25	1.80 ± 0.27	0.45	0.799
Volume of daughter tuber (mm^3^)^1^	272.66 ± 25.97^a^	246.41 ± 41.94^a^	109.67 ± 12.29^b^	25.88	<0.001
Dry biomass of a daughter tuber (g)^2^	17.88 ± 2.83^a^	12.18 ± 0.82^a^	4.58 ± 0.55^b^	32.58	<0.001
Total dry biomass of daughter tubers per plant (g)^2^	21.92 ± 3.47^a^	16.09 ± 1.32^a^	5.60 ± 0.86^b^	27.02	<0.001
Total dry biomass of mother tubers per plant (g)^2^	8.04 ± 1.03^a^	7.85 ± 0.79^a^	1.87 ± 0.21^b^	40.29	<0.001
Total belowground dry biomass (including daughter and mother tubers) per plant (g)^2^	29.97 ± 4.39^a^	23.95 ± 1.83^a^	7.86 ± 0.95^b^	36.14	<0.001
Total above ground dry biomass per plant (g)^2^	23.61 ± 2. 90^a^	21.95 ± 1.46^a^	9.95 ± 1.05^b^	16.22	<0.001
Ratio of dry biomass of belowground and aboveground parts^2^	1.34 ± 0.12^a^	1.13 ± 0.07^a^	0.86 ± 0.07^b^	7.00	<0.01
Ratio of dry biomass of reproductive and aerial vegetative parts^2^	0.21 ± 0.03^a^	0.27 ± 0.04^a^	0.46 ± 0.05^b^	9.61	<0.001

**Figure 3:**
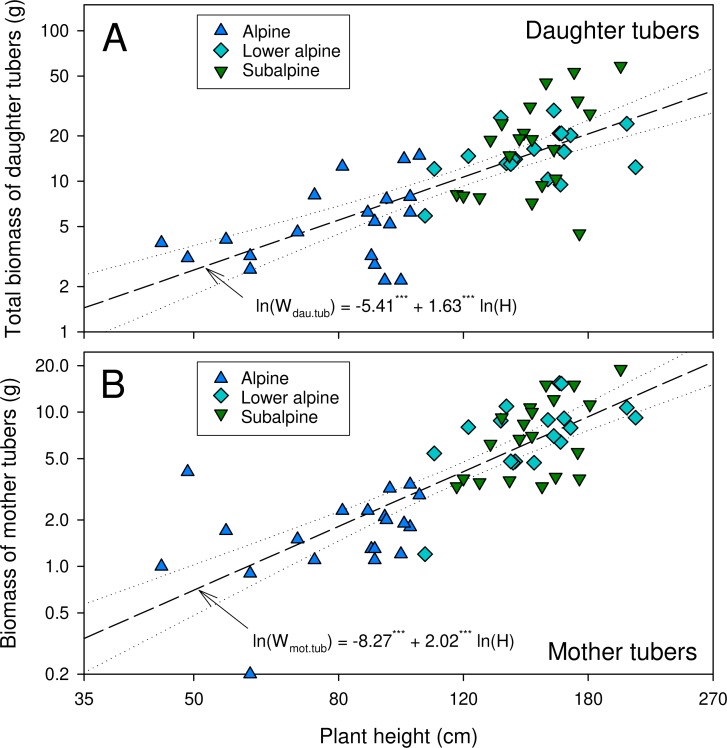
Relationship between plant height and biomass of daughter tubers (A) and mother tubers (B) of *A. spicatum*. Level of significance:***: *p* < 0.0001.

The number of daughter tubers per individual tended to be higher in higher-elevation populations though the difference was not statistically significant (Table 2). The dry mass of daughter tubers, and that of below- and aboveground plant parts were significantly lower in the alpine than in the other populations (Table 2). In lower alpine and subalpine populations, the average biomass of belowground plant parts exceeded that of the aboveground parts. The dry biomass ratios for plant parts below and above ground, and for aerial vegetative and reproductive parts, varied significantly among the studied populations (Table 2). The dry biomass ratios for plant parts below and above ground were highest for plants from the subalpine population, and the ratio of dry biomass of reproductive and aerial vegetative parts was highest in the alpine population (Table 2). The double-logarithmic allometric model revealed a significant relationship between plant height and total dry mass of tubers (both daughter and mother tubers) (*P* < 0.0001, Fig. 3). Overall, compared to the subalpine and lower alpine populations, the alpine population was generally characterized by stunted growth, low biomass, fewer and smaller leaves and fewer and smaller reproductive structures.

**Table 3:** Variation in reproductive performance of *A. spicatum* among three populations in Annapurna Conservation Area, Central Nepal. Values are mean ± SE (*n* = 225). ^∗^Population comparisons were based on either ^1^Kruskal-Wallis tests or ^2^one-way ANOVA. Values for each trait among the populations with same superscript letter do not vary significantly at *p* = 0.05 level based on multiple comparison tests after ^1^Kruskal Wallis and ^2^ANOVA.

Reproductive performance traits	Subalpine (3,000–3,300 m)	Lower alpine (3,400–3,700 m)	Alpine (3,800–4,200 m)	*χ*^2^∕*F* value*	*P*-value
Fresh weight of fruit and seed (g)^1^	0.45 ± 0.01^a^	0.55 ± 0.02^b^	0.57 ± 0.01^b^	20.98	<0.0001
Dry weight of fruit and seed (g)^1^	0.08 ± 0.004^a^	0.09 ± 0.004^ab^	0.1 ± 0.003^b^	9.64	0.008
Volume of fruit (cm^3^)^1^	18.91 ± 0.75^a^	19.19 ± 0.49^a^	14.41 ± 0.33^b^	51.58	<0.0001
No. of seeds per fruit^1^	37.21 ± 1.76^a^	47.16 ± 1.95^b^	36.63 ± 2.59^a^	17.85	<0.0001
No. of seeds per unit volume of fruit^1^	2.08 ± 0.10^a^	2.44 ± 0.07^b^	2.60 ± 0.18^ab^	7.74	0.0208
Dry weight of seeds per fruit (g)^1^	0.03 ± 0.002	0.03 ± 0.003	0.04 ± 0.003	1.34	0.511
Seed mass of 50 seeds (g)^2^	0.04 ± 0.004^a^	0.04 ± 0.004^a^	0.06 ± 0.002^b^	5.69	0.006
Seed viability (%)^2^	16.07 ± 0.99^a^	17.14 ± 1.05^a^	22.5 ± 0.64^b^	10.69	<0.0001
Fecundity	2.59	2.71	2.9		

The volume of fruits varied significantly among the populations (*χ*^2^ = 51.58, *P* < 0.0001) with highest mean value (19.19 ± 0.49 cm^3^) for the lower alpine population and lowest mean (14.41 ± 0.33 cm^3^) for the alpine population (Table 3). We found similar results for the number of seeds per fruit (*χ*^2^ = 17.85, *P* < 0.0001). The number of seeds per unit volume of fruit was found to vary significantly among the three populations with highest mean value for the alpine population (*χ*^2^ = 7.74, *P* = 0.0208). Similarly, we found higher seed mass per 50 seeds in the alpine than in the lower alpine and subalpine populations (*F* = 5.69, *P* = 0.006) (Table 3). Seed viability and fecundity were highest in the alpine and lowest in the subalpine population (Table 3). The seed viability showed statistically significant differences among the populations (*F* = 10.69, *P* < 0.0001).

### Response of density of different life stages to environmental variables (mixed ZIP models)

**Figure 4:**
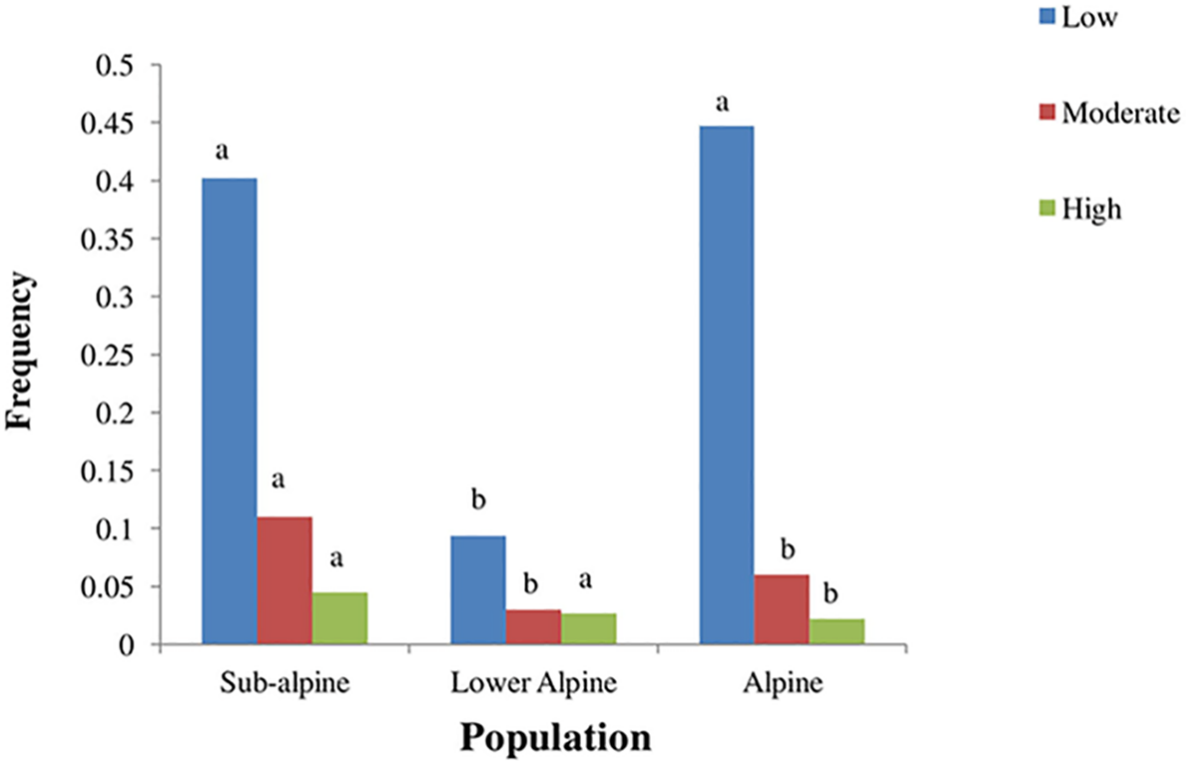
Frequency of different levels of anthropogenic disturbances in three populations of *A. spicatum* in Annapurna Conservation Area, Central Nepal. Mean of animal droppings, trampling, harvesting and fire intensity. Scale: 0–1 = low, 2 = moderate, 3–4 = high. Bars with same letter for each disturbance class among populations do not vary significantly at *p* = 0.05 level based on multiple comparison test after Kruskal Wallis.

The three studied populations varied in substrate and topographic conditions (Table 4). The subalpine population was located in herb-dominated pasture with loamy soil. The lower alpine population was in a shrub dominated pasture with high bryophyte, litter and scree cover. It had sandy loam soil. The alpine population was found in a grass-dominated pasture with silty soil. The subalpine population was affected more by anthropogenic pressure compared to the other two populations (Fig. 4).

**Table 4:** Summary of environmental variables (substrate and topographic) characterizing the three study populations of *A. spicatum* in Annapurna Conservation Area, Central Nepal. Estimates for substrate and topographic variables are specified as mean ± SE; for topographic variables ranges are also stated.

Substrate variables	Subalpine (3,000–3,300 m)	Lower alpine (3,400–3,700 m)	Alpine (3,800–4,200 m)
Shrub cover (%)	7.74 ± 1.23	11.28 ± 2.02	0.78 ± 0.51
Herb cover (%)	62.4 ± 2.51	60.2 ± 1.96	49.44 ± 3.21
Grass cover (%)	6.92 ± 1.04	8.43 ± 1.19	30.5 ± 2.89
Bryophyte cover on soil (%)	6.02 ± 0.41	7.96 ± 0.76	5.1 ± 0.46
Lichen cover on soil (%)	1.18 ± 0.10	0.89 ± 0.10	1.06 ± 0.11
Litter cover (%)	6.07 ± 0.45	11.45 ± 1.15	7.56 ± 0.80
Solid rock cover (%)	16.62 ± 2.59	7.65 ± 1.10	5.34 ± 1.15
Scree cover (%)	0.00 ± 0.00	0.63 ± 0.20	0.00 ± 0.00
Bare ground cover (%)	0.80 ± 0.28	2.78 ± 0.41	1.00 ± 0.27
Lichen cover under vascular plants (%)	1.05 ± 0.10	0.88 ± 0.13	1.04 ± 0.14
Bryophyte cover under vascular plants (%)	9.63 ± 0.74	11.72 ± 1.01	5.92 ± 0.65
Bryophyte cover on rock (%)	24.93 ± 3.34	17.92 ± 2.67	7.49 ± 1.66
Lichen cover on rock (%)	2.37 ± 0.50	1.06 ± 0.15	4.73 ± 1.18
Bare rock cover (%)	20.88 ± 3.15	41.86 ± 3.84	22.22 ± 3.70
Topographic variables			
Elevation (m a.s.l.)	3,204.16 ± 9.74 (3,000–3,300)	3,584.16 ± 11.15 (3,400–3,700)	3,968.66 ± 11.80 (3,800–4,200)
Slope (degrees)	24.5 ± 1.37 (3–55)	19.25 ± 1.03 (3–39)	11.94 ± 0.94 (3–40)
Relative radiation index (RRI)	0.85 ± 0.01 (0.71–0.99)	0.88 ± 0.01 (0.78–0.99)	0.85 ± 0.01 (0.59–0.96)

Mixed zero-inflated Poisson (mixed ZIP) regression models revealed that harvesting and herb cover had significant negative effects on seedling density. For juvenile density, harvesting had a significant negative effect. The other variables tested did not show significant effects on seedling and juvenile density. In the case of adult stages (both vegetative and reproductive adults) population showed a significant effect on density. The mixed ZIP model analysis further showed that, compared to the alpine population and taking cover and disturbance variables into account, the subalpine population exhibited a significantly lower density of vegetative and reproductive adult stages. Interestingly, animal droppings and fire had a significant positive effect on adult density (Table 5). The zero–inflation model component was significant for juvenile and adult reproductive stages but no covariates showed any significant effect on this component.

**Table 5:** Mixed Zero-Inflated Poisson regression models for the density (m^−2^) of seedling, juvenile, adult vegetative and adult reproductive stages of *A. spicatum*. Densities of different stages were modeled as functions of population, relative radiation index (RRI), shrub cover (%), herb cover (%) and disturbance (animal droppings, trampling, harvesting and fire, which were assessed using an ordinal 0–4 scale). Zi is the intercept in the zero-inflation component; all other parameters refer to the count component of the model. Parameter estimates with standard errors in brackets for Full average and Conditional average models.

		ZI component	Count component									
	Average model	Zi	Intercept	Lower alpine	Subalpine	Shrub cover	Herb cover	RRI	Harvesting	Trampling	Animal droppings	Fire
Seedling	Full	−0.382	−1.703	−0.185	−0.303	0.003	−0.008	1.247	−0.955^**^	0.011	0.022	0.007
		(1.003)	(2.109)	(0.410)	(0.555)	(0.006)	(0.007)	(2.349)	(0.332)	(0.131)	(0.072)	(0.063)
	Conditional	−0.382	−1.703	−0.185	−0.303	0.010	−0.012^*^	3.491	−0.955^**^	0.053	0.185	0.081
		(1.003)	(2.109)	(0.411)	(0.555)	(0.008)	(0.006)	(2.759)	(0.332)	(0.281)	(0.116)	(0.191)
Juvenile	Full	0.941^***^	−0.131	0.083	−0.620	0.001	−0.000	0.248	−0.536^*^	−0.187	0.044	0.001
		(0.280)	(1.110)	(0.380)	(0.573)	(0.005)	(0.003)	(1.188)	(0.252)	(0.269)	(0.093)	(0.086)
	Conditional	0.941^**^	−0.131	0.083	−0.620	0.011	0.000	1.390	−0.561^*^	−0.369	0.182	0.007
		(0.280)	(1.109)	(0.380)	(0.573)	(0.009)	(0.005)	(2.510)	(0.229)	(0.276)	(0.102)	(0.189)
Adult	Full	0.121	0.206	0.152	−1.685^**^	−0.000	0.000	0.138	−0.097	−0.104	0.123	−0.029
Vegetative		(0.198)	(0.589)	(0.215)	(0.375)	(0.001)	(0.002)	(0.630)	(0.125)	(0.155)	(0.088)	(0.085)
	Conditional	0.121	0.206	0.152	−1.685^**^	−0.002	0.003	1.749	−0.173	−0.183	0.165^**^	−0.047
		(0.198)	(0.589)	(0.215)	(0.375)	(0.007)	(0.003)	(1.478)	(0.121)	(0.167)	(0.060)	(0.104)
Adult	Full	−1.007^**^	0.035	0.171	−0.826^**^	0.000	−0.000	0.044	−0.025	−0.046	0.107	0.178*
reproductive		(0.312)	(0.329)	(0.181)	(0.235)	(0.00)	(0.000)	(0.334)	(0.059)	(0.096)	(0.064)	(0.082)
	Conditional	−1.007^**^	−0.035	0.171	−0.826^***^	0.004	−0.000	1.485	−0.088	−0.099	0.124^*^	0.186^*^
		(0.312)	(0.329)	(0.181)	(0.235)	(0.004)	(0.002)	(1.255)	(0.083)	(0.121)	(0.052)	(0.074)

Notes.

Levels of significance: *: *p* < 0.05; **: *p* < 0.01; ***: *p* < 0.001.

## Discussion

### Variation in population density and structure

The mean density of *A. spicatum* in the populations examined is comparable with previous findings. For example, densities of 0.34 (Kala, 2005) to 3.71 (Nautiyal et al., 2002) plants/m^2^ in *A. balfourii*, 2.57 (Nautiyal et al., 2002) to 3.1 (Semwal et al., 2007) plants/m^2^ in *A. heterophyllum*, 2.7 plants/m^2^ in *A. rotundifolium* (Kala, 2005), and 2 plants/m^2^ in *A. violaceum* (Kala, 2000; Kala, 2005) have been reported from the Indian Himalayas. However, much higher densities have also occasionally been observed. For example, Nautiyal et al. (2002) recorded a density of 6.7 plants/m^2^ in *A. violaceum* in Gharwal Himalaya, India, which was attributed to limited human exploitation due to habitat inaccessibility. Similarly, Shrestha & Jha (2010) reported a mean density of 7.3 plants/m^2^ in *A. naviculare* in Manang, Nepal, which was described as a result of subjective sampling due to the patchy distribution of the species.

In the high mountain ecosystem, elevation represents a complex gradient closely correlated with numerous other environmental variables, such as temperature, precipitation, soil texture and nutrients, substrate stability, and disturbance (Auerbach & Shmida, 1993; Ramasay & Oxley, 1997). All these factors affect plant life in a number of ways. Our mixed ZIP model showed that the subalpine population exhibited a significantly lower density of adult vegetative and adult reproductive plants than did the lower alpine and alpine populations. The higher plant density in lower alpine and alpine populations might partly be due to lower human pressure (Fig. 4) in these habitats. In addition, the smaller size of individuals in higher-elevation populations also makes it possible for a higher number of individuals to share a given amount of space. With increasing elevation the number of competing species of similar or larger size decreases. This causes a small area to hold a large number of individuals, thus increasing the density. The shift from an environment characterized by high degree of competition to a more facilitative environment favors this higher density (Liancourt et al., 2017). Bosch & Waser (1999) found that the pollinator visitation is higher in dense populations of plants than in sparse populations, which may further increase seed set and ultimately the population density.

The low proportions of younger life stages (seedlings and juveniles) and correspondingly high proportion of adults (vegetative and reproductive) in the studied populations clearly indicate that the recruitment potential of *A. spicatum* is generally rather low. The recruitment was further reduced in the subalpine population. The small seed size, and low seed viability and fecundity recorded in the subalpine population compared to those of the lower alpine and alpine populations support that the subalpine population is suffering from lack of sufficient natural regeneration, consequently lowering the population density. Studies have shown that disturbance characteristics determine the success of different reproductive strategies (Chambers, 1995). Anthropogenic disturbance, like harvesting and trampling can reduce seed viability and fecundity in plants from mountain ecosystems (Tonnabel et al., 2017) and thereby reduce the population density.

### Response of density of different life stages to environmental variables

The mixed ZIP model showed significant negative impact of harvesting on both seedling and juvenile plant density, and population (elevation) had a significant negative effect on adult density. Based on conversations with collectors and key informants, it appears that traditional healers prefer to harvest *A. spicatum* at younger stages, believing that relatively young tubers have high medicinal efficacy. However, commercial collectors prefer mostly the large adult plants. Removal of the entire plant before seed maturation reduces the possibility of seed development for future regeneration (Sheldon, Balik & Laird, 1997). The negative effects of such damages caused by collectors may be further enhanced by infrequent sexual regeneration and possibly also by limited availability of suitable sites for regeneration in subalpine and alpine grasslands (Austrheim & Eriksson, 2001). The mixed ZIP model analysis further showed that herb cover had a significant negative impact (at the 10% level) on the density of seedlings, which may be a consequence of herb cover reducing the space available for seedling establishment, increasing the competition and thereby affecting growth and development.

Although frequent trampling by sheep, wild animals and humans damage plants and prevents them from flowering, developing seeds to maturity, and dispersing their seeds (Chardon et al., 2018), and seedlings of *A. spicatum* may be particularly sensitive in this regard, as they have soft stems with only one to two leaves, we found positive effects of some disturbances on density of adults. Mixed ZIP model revealed positive significance of animal droppings and fire on adult (both vegetative and reproductive) density. Direct observation also support the result as the *A. spicatum* individuals seemed to show preference for growing in the proximity of cattle sheds where the soil had higher concentration of animal droppings. Ohlson & Grønli (2006) also reported a significant positive effect on life history parameters in *A. septentrionale* growing in experimental plot supplied with higher concentrations of nutrients. The tolerance of adult stages of *A. spicatum* to fire in the present study is comparable with the findings of Uys, Bond & Everson (2004) who showed the tolerance of many forb species to fire.

### Variation in plant size and reproductive performance

*A. spicatum* has developed an astonishing range of life history traits in response to highly specific ecological environments in subalpine, lower alpine and alpine habitats. A high degree of variation with respect to different traits was found among the populations (Table 2). The decreasing trend of plant height, stem girth, leaf area, length of floral axis and plant biomass observed in plants from subalpine to alpine populations (Table 2) showed that the size of individuals generally tends to decrease with increasing elevation. *A. spicatum* in the subalpine (lower elevation) population exhibited better performance in terms of high vegetative vigor and production of higher number of flowers and fruits per individual. Shrestha & Jha (2010), studying *A. naviculare,* also reported higher vigor for the individuals examined in a lower elevation population. Plants may perform better simply because of favorable abiotic conditions (Billings, 1973; Körner, 2003), such as temperature and moisture (Körner, 2003). Plant performance is also enhanced in habitats with better abiotic conditions through facilitation (positive interactions), a common phenomenon at high altitudes, which helps plants to cope with the harsh environment (Callaway et al., 2002). Thus, the higher vegetative vigor of *A. spicatum* observed at lower elevation is presumably related to the presence of appropriate abiotic conditions in which plants exhibit enhanced growth (Shrestha & Jha, 2010). The harsh environmental conditions at higher elevation generally impose constraints on plant growth (Körner, 2003). Similar trends of decreasing plant vigor with increasing elevation have been observed in other herbs, such as *Silene acaulis*, in which disturbance has been described as beneficial for the performance of plants at lower elevation (Chardon et al., 2018).

The decreasing number of flowers and fruits set that we observed along the elevation gradient is likely related to plant size but could also be due to the time of flowering, which is influenced by the ambient temperature and the timing of snow melt (Kudo & Hirao, 2006). Flower production is often correlated with plant size, which generally increases with resource availability and decreases with increasing plant density (Weiner & Thomas, 1986). Moreover, flower and fruit production are shaped by the interactions between abiotic and biotic environment (Ma et al., 2010; Agren, Ehrlén & Solbreck, 2008). For example, population density negatively affects fruit production of individual plants through its effect on flower number (Agren, Ehrlén & Solbreck, 2008). Disturbances like trampling frequently break the inflorescence, and repeated disturbances year after year also reduce the reproductive potential of the individuals, thus limiting the production of flowers and fruits (Chardon et al., 2018).

Like many high-elevation plants (e.g., *Arenaria kansuensis, Rhodiola quadrifida, Tribulus terrestris* etc.), *A. spicatum* also allocated a large amount of biomass to its underground structure and, as a result, aboveground reproductive organs are reduced and roots are enlarged (Ma et al., 2010). A high proportion of belowground biomass has often been interpreted as an adaptive response to severe environmental conditions (Webber & May, 1977). Particularly for high-elevation plants, subterranean organs are dedicated to produce annual aerial parts shortly after snow melt and, thus, high elevation plants are found to invest more in belowground parts as an adaptive strategy.

Despite the lower number of flowers and fruits set per individual in alpine populations, in terms of reproductive allocation, these plants performed far better than subalpine and lower alpine populations. The ratio of dry biomass of reproductive to aerial vegetative parts was higher in plants from the alpine population than in plants from subalpine and lower alpine populations, and this clearly indicates that plants from very high elevation invest more in reproduction. The subalpine population had larger fruits than the two other populations and, as a result, they produced higher number of seeds per fruit. Contrary to this, though producing the smallest number of fruits, the plants from the alpine population, compared to the two other populations from lower elevation, set higher number of seeds per unit volume of fruits, and these were seeds with a higher seed mass. Seed production by species from high elevations depend on environmental conditions (short, cool growing seasons) and is temporally and spatially variable (Chambers, 1995). The increasing seed mass with increasing elevation can be explained by more resources being allocated to the individual seed in alpine populations, thereby increasing their viability and fecundity to cope with a stressful environment. The increase in seed mass together with a reduced number of seeds per individual in populations from higher elevations may indicate that *A. spicatum* allocates more resources to the seeds and may reflect a strategy that helps increasing fitness and long-term population persistence in harsh alpine environments.

## Conclusion

Human disturbance and topographical factors are related to plot-level microclimatic conditions, which contribute to the variation in density and the studied life history traits. Among the environmental variables tested, harvesting, animal droppings and fire appeared to be the most important (proxy) factors affecting the density of *A. spicatum*. The population at the lowest elevation was exposed to the highest anthropogenic pressure, the level of which decreased with increasing elevation. The prevailing harvesting pattern was destructive as it involved uprooting of the whole plant and was probably a main reason for the lower recruitment and reduced density in the subalpine population. The enhanced vegetative performance of adults and the higher number of flowers and fruits set per reproductive individual in the subalpine population cannot compensate for the lower recruitment and loss of younger plants due to high disturbance. The lower recruitment in the subalpine population was presumably also related to reduced seed mass, and decreased seed viability. In terms of reproductive effort (higher seed set per unit fruit volume, higher seed mass, and higher seed viability and fecundity), the alpine populations were performing better. This confirms that alpine plants develop self-sustaining adaptive strategies by increasing their resource allocation in reproductive parts, especially in seeds, to cope with the harsh environment. As the subalpine population was suffering from reduced reproductive performance, the persistence and growth of *A. spicatum* at low elevations will depend on how effectively management will address the protection of younger life stages and control over-harvesting and premature harvesting of tubers. Although the adult individuals were found tolerant to animal droppings and fire, the viability of *A. spicatum* populations not only depends on controlling over harvesting and premature harvesting but also on mitigating other common anthropogenic disturbances, like grazing and trampling, to ensure completion of flowering, fruiting and dispersal of viable seeds. We therefore recommend that when formulating management guidelines, measures aiming to mitigate such anthropogenic disturbances should be considered. As science-based harvesting and conservation strategies have not yet been developed for *A. spicatum* and other Himalayan MAPs, and harvest and trade are conducted on ad hoc basis, we hope that this study will contribute to the conservation of MAPs by helping government authorities to formulate scientifically informed conservation management strategies for *A. spicatum* and other similar MAPs.

##  Supplemental Information

10.7717/peerj.7574/supp-1 Dataset S1 Variation in life history traits (vegetative traits and reproductive outputs) of *Aconitum spicatum* among three populations in Annapurna Conservation Area, Central Nepal *Population comparisons were based on either ^1^Kruskal-Wallis tests or ^2^one-way ANOVA.

10.7717/peerj.7574/supp-2 Dataset S2 Variation in reproductive performance of *Aconitum spicatum* among three populations in Annapurna Conservation Area, Central Nepal Values are mean ± SE (*n* = 225).
*Population comparisons were based on either ^1^Kruskal-Wallis tests or ^2^one-way ANOVA.

10.7717/peerj.7574/supp-3 Dataset S3 Mixed Zero-inflated Poisson regression models for the density (m^−2^) of seedling, juvenile, adult vegetative and adult reproductive stages of *Aconitum spicatum* Densities of different stages were modeled as functions of population, relative radiation index (RRI), shrub cover (%), herb cover (%) and disturbance (animal droppings, trampling, harvesting and fire, which were assessed using an ordinal 0–4 scale). Zi is the intercept in the zero-inflation component; all other parameters refer to the count component of the model. Parameter estimates with standard errors in brackets for Full average and Conditional average models.

10.7717/peerj.7574/supp-4 Appendix S1 List of all sets of models prepared using glmmTMB package during analysis

10.7717/peerj.7574/supp-5 Appendix S2 Model selection table for Seedling, Juvenile, adult vegetative and adult reproductive stages

## Additional Information and Declarations

### Field Study Permissions

The following information was supplied relating to field study approvals (i.e., approving body and any reference numbers): Field works were approved by the Department of National Park and Wildlife Conservation, Nepal (ref. No. 93/070/071).

### Data Availability

The following information was supplied regarding data availability: Raw data are available in Datasets S1–S3.
