# Supplementary material for: Plant density and life history traits of Aconitum spicatum in North-central Nepal: effects of elevation and anthropogenic disturbances
Source: PeerJ. 2019 Sep 10;7:e7574. doi: 10.7717/peerj.7574 (PMC6743441; doi:10.7717/peerj.7574)
Supplement: Appendix S1 [file peerj-07-7574-s004.docx]

**Set of models prepared using glmmTMB during analysis**

**Model 1**

Density _ij_ (of a particular stage of *A. spicatum*) = a + (1|Plot)

**Model 2**

Density _ij_ (of a particular stage of *A. spicatum*) = a + b(Population) (1|Plot)

**Model 3**

Density _ij_ (of a particular stage of *A. spicatum*) = a + b(Population)+ c_1_Herb cover_ij_+ (1|Plot)

**Model 4**

Density _ij_ (of a particular stage of *A. spicatum*) = a + b(Population)+ c_1_Herb cover_ij_+ c_2_ Shrub cover _ij_+ (1|Plot)

**Model 5**

Density _ij_ (of a particular stage of *A. spicatum*) = a) + b(Population)+ c_1_Herb cover _ij_ + c_2_ Shrub cover _ij +_  c_3_Harvesting _ij_+ (1|Plot)

**Model 6**

Density _ij_ (of a particular stage of *A. spicatum*) = a + b(Population)+ c_1_Herb cover _ij_ + c_2_Harvesting _ij_+ (1|Plot)

**Model 7**

Density _ij_ (of a particular stage of *A. spicatum*) = a + b(Population)+ c_1_ Shrub cover _ij+_ c_2_Harvesting _ij_  _j_+ (1|Plot)

**Model 8**

Density _ij_ (of a particular stage of *A. spicatum*) = a + b(Population) + c_1_ RRI + (1|Plot)

**Model 9**

Density _ij_ (of a particular stage of *A. spicatum*) = a + b(Population) + c_1_Harvesting + (1|Plot)

**Model10**

Density _ij_ (of a particular stage of *A. spicatum*) = a + b(Population) + c_1_Herb_cover _ij_ + c_2_ Shrub cover _ij +_ c_3_ Harvesting _ij_  + c_4_RRI _ij_ + (1|Plot)

**Model 11**

Density _ij_ (of a particular stage of *A. spicatum*) = a + b(Population) + c_1_Harvesting _ij_  + c_2_RRI _ij_ + (1|Plot)

**Model12**

Density _ij_ (of a particular stage of *A. spicatum*) = a + (1|Plot) + b(Population) + c_1_Herb_cover _ij_ + c_2_RRI _ij_

**Model13**

Density _ij_ (of a particular stage of *A. spicatum*) = a + b(Population) + c_1_Shrub_cover _ij_ + c_2_RRI _ij_ + (1|Plot)

**Model14**

Density _ij_ (of a particular stage of *A. spicatum*) = a + b(Population) + c_1_Herb_cover _ij_ + c_2_Harvesting _ij_ + c_3_RRI _ij_ + (1|Plot)

**Model15**

Density _ij_ (of a particular stage of *A. spicatum*) = a + b(Population) + c_1_Shrub_cover _ij_ + c_2_Harvesting _ij_ + c_3_RRI _ij_ + (1|Plot)

**Model16**

Density _ij_ (of a particular stage of *A. spicatum*) = a + b(Population) + + c_1_RRI _ij +_c_2_Herb_cover _ij_ + c_3_ Shrub cover _ij +_ c_4_ Harvesting _ij_  + c_5_Trampling _ij +_ c_6_ Animal drooping _ij+_  c_7_ Fire _ij_  + (1|Plot)

**Model17**

Density _ij_ (of a particular stage of *A. spicatum*) = a + b(Population) + c_1_ Harvesting _ij_  + c_2_Trampling _ij +_ c_3_ Animal drooping _ij+_  c_4_ Fire _ij_  + (1|Plot)

**Model18**

Density _ij_ (of a particular stage of *A. spicatum*) = a + b(Population) + c_1_ Harvesting _ij_  + c_2_Trampling _ij +_ c_3_ Animal drooping _ij+_  c_4_ Fire _ij_  + c_6_ Herb _ij_  + (1|Plot)

**Model19**

Density _ij_ (of a particular stage of *A. spicatum*) = a + b(Population) + c_1_Herb_cover _ij_  _+_ c_2_ Harvesting _ij_  + c_3_Trampling _ij +_ c_4_ Animal drooping _ijj_ + (1|Plot)

**Model20**

Density _ij_ (of a particular stage of *A. spicatum*) = a + b(Population) + c_1_Herb_cover _ij_ + c_2_ Harvesting _ij_  + c_3_Trampling _ij_ + (1|Plot)

**Model21**

Density _ij_ (of a particular stage of *A. spicatum*) = a + b(Population) + c_1_Harvesting _ij_  + c_2_Trampling _ij_ + (1|Plot)

**Model22**

Density _ij_ (of a particular stage of *A. spicatum*) = a + b(Population) + c_1_Fire _ij +_ c_2_ Harvesting _ij_  + c_3_Trampling _ij_ + (1|Plot)

**Model23**

Density _ij_ (of a particular stage of *A. spicatum*) = a + b(Population) + c_1_Animal drooping _ij_ + c_2_ Trampling _ij +_ c_3_ Fire _ij_  + (1|Plot)

**Model24**

Density _ij_ (of a particular stage of *A. spicatum*) = a + b(Population) + c_1_Animal drooping _ij_ + c_2_ Fire _ij_ + (1|Plot)

**Model25**

Density _ij_ (of a particular stage of *A. spicatum*) = a + b(Population) + c_1_Trampling _ij_ + c_2_ Fire + (1|Plot)

**Model26**

Density _ij_ (of a particular stage of *A. spicatum*) = a + b(Population) + c_1_Harvesting _ij_  + c_2_Fire_ij_ + (1|Plot)

Where, a, b(Population) and c_1_…c_13_ are fixed model parameters, i=1…66 is the plot (random effect) and j=1…5 is the sub-plot. Population has three categories (subalpine, lower alpine and alpine). Density is expressed as the number of individuals in a given stage category counted within a 1 m^2^ sub-plot.
